# A Regulator SNP Affecting the Transcription Factor *SP1* Binding Site in the *PIK3R3* Gene Contributes to Noise‐Induced Hearing Loss Risk

**DOI:** 10.1155/humu/7010704

**Published:** 2026-10-05

**Authors:** Long Miao, Jiaxuan Zhou, Man Qu, Yu Xue, Xiuli Zhao, Xiaoqin Li, Yang Wang, Shuang Qiu

**Affiliations:** ^1^ School of Nursing and School of Public Health Yangzhou University Yangzhou China yzu.edu.cn; ^2^ Key Laboratory of Environmental Medicine Engineering Ministry of Education School of Public Health Southeast University Nanjing China seu.edu.cn; ^3^ Department of Radiology and Functional Examination Nanjing Prevention and Treatment Center for Occupational Diseases Nanjing China; ^4^ Department of Occupational Health Yangzhou Center for Disease Control and Prevention Yangzhou China; ^5^ Yangzhou Hospital of Traditional Chinese Medicine Affiliated to the School of Clinical Chinese Medicine Yangzhou University Yangzhou China yzu.edu.cn

**Keywords:** noise-induced hearing loss, *PI3K/AKT* pathway, *PIK3R3*, single nucleotide polymorphisms, *SP1*

## Abstract

**Background:**

Noise‐induced hearing loss (NIHL) is a key occupational hazard, and its specific pathogenic mechanism is not fully elucidated. Here, we performed this study to explore the potential role of single nucleotide polymorphisms (SNPs) located in the *PI3K/AKT* pathway in human susceptibility to NIHL.

**Methods:**

A case‐control study including 688 NIHL patients and 667 normal subjects was conducted. Genotype analysis was tested by multiplex polymerase chain reaction (PCR) with next generation sequencing. Luciferase assays were conducted to explore the effect of *SP1* on *PIK3R3* promoter activity. siRNA and cell transfection were used to determine the regulatory effect of *SP1/PIK3R3* on HEI‐OC1 cells. qRT‐PCR and Western blotting were used to determine the expression of genes and proteins related to *PI3K/AKT*, autophagy, and apoptosis. Cell counting kit 8 (CCK‐8) assay was used to detect cell viability.

**Results:**

Subjects with *PIK3R3* rs7536272 GG genotype were more sensitive to NIHL than those with the AA/AG genotypes and had a lower expression level of *PIK3R3*. Luciferase assay indicated that rs7536272 regulated *PIK3R3* expression by binding to *SP1*. Knockdown of *SP1* by siRNA and transfection into HEI‐OC1 cells inhibited the *PI3K/AKT* pathway, indicated by the decreased phosphorylation levels of *PI3K* and *AKT*. Concurrently, *SP1* deficiency promoted autophagy, as evidenced by an elevated *LC3-II/LC3-I* ratio and induced apoptosis, characterized by the reduction of *Bcl2* and the increase of *Bax* and *caspase-3*. Overexpression of *PIK3R3* partially restored the decreased activity of *PI3K/AKT* pathway and attenuated the enhanced autophagy and apoptosis caused by *SP1* deficiency. CCK‐8 assay revealed that *SP1* knockdown led to a time‐dependent decrease in cell viability, which was significantly rescued by the overexpression of *PIK3R3*.

**Conclusion:**

All findings demonstrate that rs7536272 influences NIHL susceptibility by regulating *PIK3R3* expression in an allele‐specific manner by binding to *SP1*. *SP1* inhibits autophagy and apoptosis by activating *PIK3R3*‐mediated *PI3K/AKT* pathway.

## 1. Introduction

Noise‐induced hearing loss (NIHL) is the most common sensorineural hearing loss caused by long‐term exposure of the auditory system to noise environments in the workplaces and is a critical occupational hazard that has become a focus of auditory science and even public health research [1]. Research data showed that the global disability adjusted life years (DALYs) increased from 3.3 million to 6 million attributing to occupational noise inducing NIHL from 1990 to 2017 [2]. Globally, occupational noise causes disabling hearing loss in 16% of adults [3]. Currently, more than 1.5 billion people worldwide have different degrees of hearing loss, and the number will increase to approximately 2.5 billion by 2050, suggesting the urgency and importance of NIHL prevention and control [4]. Recent research reported that NIHL was more prevalent in low‐ and middle‐income countries, with prevalence rates ranging from 18% to 67% [5]. Additionally, in China, approximately 25.14% of workers were engaged in productive activities in workplaces with noise exposure levels exceeding the national allowable standard of 85 dB(A), which poses a serious threat to workers′ physical and mental health and production safety [6]. Previously, we performed a cross‐sectional study on Chinese noise‐exposed workers and found that the prevalence of NIHL was up to 24.38%, indicating that the noise‐exposed workers had a high risk of developing NIHL [7].

The pathogenic mechanism of NIHL is the focus and difficulty of this research field. NIHL is a complex disease caused by both environmental and individual factors, which affects the entire life cycle and alters the trajectory of hearing ([8]). Early studies have shown that the mechanism of inner ear damage caused by noise is attributed to the pathological changes resulting from the physical mechanical impact of sound wave [9]. The impact of individual susceptibility differences caused by genetic variations on NIHL more than 50% [10]. Several known single nucleotide polymorphisms (SNPs) in the antioxidant genes, potassium channel‐related genes, and heat shock protein gene were associated with susceptibility to NIHL [11]. Current studies only explained a small amount of the risk of NIHL, so it is necessary to further discover and explore new NIHL pathogenic genes and genetic variants.

The phosphoinositide 3‐kinase (*PI3K*)/protein kinase B (*AKT*) signaling pathway was reported to participate in a range of cellular and biological processes, including gene expression, protein synthesis, cell survival, and apoptosis [12]. Recent evidence points to *PI3K/AKT* regulating the autophagic response of body under a variety of physiological and pathophysiological conditions [13]. The *PI3K/AKT* signaling pathway was strongly associated with NIHL. It was shown by animal model that *PI3K/AKT* signaling blockade can increase NIHL susceptibility ([14]). Also, available evidence showed that the *PI3K/AKT* signaling has a potential role in preventing NIHL by calpain inhibitor in CBA/J mice model [15]. Previously, our population and animal studies suggested that the aberrant expression of major genes in the *PI3K/AKT* pathway was closely related to NIHL [16, 17].

Considering the important role of *PI3K/AKT* pathway in hearing loss especially caused by noise and ototoxic drugs, we wonder whether there are some functional SNPs in human *PI3K/AKT* pathway genes contributing to the individual variations in NIHL. There is no data available on this relationship so far. Here, we conducted this study to investigate the potential role and mechanism of functional SNPs within the key genes including *PIK3CA*, *PIK3R1*, *PIK3R3*, and *AKT1* in the *PI3K/AKT* molecular signaling pathway on NIHL susceptibility.

## 2. Materials and Methods

### 2.1. Subjects

In the current research, we carried out a case‐control analysis to explore the association between candidate SNPs and the susceptibility to NIHL, and to reveal the potential mechanism underlying NIHL. Overall, 688 diagnosed NIHL patients and 667 controls without hearing loss were recruited into this current study. All patients were the frontline workers of noise operations in key occupational disease monitoring enterprises in Jiangsu province between 2018 and 2020. All workers undergo annual occupational health examinations during their employment, including routine physical examinations, peripheral venous blood collection, and pure‐tone audiometry (PTA). In detail, the inclusion criteria for subjects in the study were as follows: (1) were from the Han Chinese population, (2) exposed to occupational noise for more than 3 years, and (3) only exposed to occupational noise hazard. The exclusion criteria included the following: (1) had family history of hereditary deafness, (2) had history of explosive deafness, (3) had history of head injury, (4) had history of ear disease, (5) had history of previous or current use of ototoxic drugs, and (6) no record of occupational health examination data.

This study was performed under the approval of the Ethics Committee of Zhongda Hospital of Southeast University (Approval No. 2020ZDSYLL150‐P01). Meanwhile, before this study began, written informed consent forms were obtained from each study subject.

### 2.2. Noise Exposure Level Detection in Workplace

The details of the detection of noise exposure intensity have been previously described elsewhere. [7]. In brief, noise exposure intensity in the workplaces was measured based on 8‐h continuous equivalent A‐weighted sound pressure level (L_Aeq_, 8 h) using SoundPro digital sound level meter (TSI Quest, United States). In this study, noise exposure was defined as L_Aeq_, 8 h of at least 85 dB(A). In addition, cumulative noise exposure (CNE) was used to quantify the amount of noise exposure for individuals [18].

### 2.3. PTA Examination

As we elucidated in a previous study [7], PTA was implemented by an audiologist to evaluate the binaural hearing thresholds of all subjects at different frequencies including 500, 1000, 2000, 3000, 4000, and 6000 Hz with Madsen Voyager 522 audiometer (Madsen, Taastrup, Denmark) in a soundproof chamber with background noise below 25 dB(A). In brief, to eliminate the influence of temporary threshold shift (TTS) on hearing, all subjects were demanded to get rid of noise working positions for 12–48 h before PTA tests. After the tests, the results of PTA were further corrected for age and gender according to the requirement of acoustics‐statistical distribution of hearing thresholds as a function of age (GB/T 7582‐2004). Based on the diagnostic criteria for occupational NIHL, the binaural high‐frequency (3000, 4000, and 6000 Hz) threshold on average (BHFTA) below 25 dB(A) was defined as normal hearing; however, BHFTA worse than 25 dB(A) was recognized as NIHL.

### 2.4. SNP Selection and Genotyping

Genomic DNA in the peripheral blood sample was extracted using the RelaxGene Blood DNA System (Tiangen Biotech, Beijing, China). The International HapMap Project [19], dbSNP database [20], and 1000 Genomes Project [21] were established to identify and map more common disease‐causing variants in the human genome, particularly SNPs, in order to promote understanding of the genetic basis of complex diseases and to guide genetic research related to human health and diseases. Evidence indicated that genetic impacts of many common diseases are caused by a limited number of allelic variantions, which account for 1%–5% of the population [22]. It is estimated that there are approximately 10 million SNPs loci in the human population, with an average of one locus for every 300 bases, leading to a frequency of at least 1% for both alleles [19]. Variants in the promoter and exon coding region could affect the alterations of transcription level of genes and protein structure; besides, intronic splicing variants can impact the splice sites and the binding of regulatory factors to splicing silencers or enhancers [23, 24]. To sum up, the three databases were used to search for candidate SNPs in the Chinese Han population, with a focus on four genes related to *PI3K/AKT* signaling pathway, including *PIK3CA*, *PIK3R1*, *PIK3R3*, and *AKT1*. The selection criteria were based on the following: (1) locate in the promoter region, exon region, or intron region, (2) minimum allele frequency (MAF) > 0.01 in the Chinese Han population, and (3) *r* ^2^ value of linkage disequilibrium > 0.80. Eight candidate SNPs in the four genes were identified, including one candidate SNPs in *PIK3CA*, three in *PIK3R1*, one in *PIK3R3*, and three in *AKT1*. Furthermore, these SNPs have previously been reported to be associated with a variety of other diseases in humans [25–28].

For SNP genotyping, three round multiplex PCR with next generation sequencing method was utilized to test the genotypes of selected SNPs in 688 cases with NIHL and 667 controls ([29]). Genotypic analyses of SNPs were carried out by Shanghai Biowing Applied Biotechnology Co., Ltd.

### 2.5. RNA Extraction and qRT‐PCR Analysis

RNAprep Pure Hi‐Blood Kit (Tiangen Biotech, Beijing, China) was used to efficiently extract the total RNA from peripheral blood. Next, cDNA was synthesized using the Takara PrimeScriptTM RT Master Mix assay kit (Takara, Japan). Specific primers for *PIK3R3* and *β*‐actin were designed and synthesized by Generay Biotechnology (Shanghai, China). SYBR Green Real‐time PCR Master Mix‐Plus kit (Toyobo, Japan) and Quant Studio 6 Flex system (Applied Biosystems, United States) were used to conduct qRT‐PCR analysis. Moreover, *β*‐actin was used as an internal control and the relative RNA expression of *PIK3R3* was evaluated adopting 2^−*Δ* *Δ* *C* *т*^ method.

Specific primers used in this current study were as follows: *PIK3R3* (forward: 5 ^′^‐TACAATACGGTGTGGAGTATGGA‐3 ^′^; reverse: 5 ^′^‐TCATTGGCTTAGGTGGCTTTG‐3 ^′^) and *β*‐actin (forward: 5 ^′^‐CTACCTCATGAAGATCCTCACCGA‐3 ^′^; reverse: 5 ^′^‐TTCTCCTTAATGTCACGCACGATT‐3 ^′^).

### 2.6. Bioinformatics Analysis

Available evidence showed that SNPs can influence gene transcription, mRNA stability, and protein structure [30]. SNPs in the promoter region can affect promoter activity by interfering the transcription factor binding [31]. Rs7536272 was located in the *PIK3R3* promoter region; we speculated that it may act as a significant transcription factor binding‐disrupting SNP regulating *PIK3R3* expression. Next, two online prediction databases including ChIPBase and hTFtarget were used to determine the transcription factor potentially interacting with rs7536272.

### 2.7. Cell Culture and Transfection

Mouse auditory cell line House Ear Institute‐Organ of Corti 1 (HEI‐OC1) was cultured in DMEM medium (Gibco, United States) supplemented with 10% FBS (Gibco, United States) under 10% CO_2_ and 33°C conditions.

siRNA directed knockdown of *SP1* (si‐*SP1*) and siRNA negative control (si‐NC) were synthesized by HippoBio (Huzhou, China). The *PIK3R3* expression vector was constructed by Jijiang Biotechnology Co., Ltd (Nanjing, China). HEI‐OC1 cells were transfected with si‐NC, *si-SP1*, and si‐*SP1* plus *PIK3R3* overexpression (*PIK3R3*‐OE) plasmids by Lipofectamine 2000 reagent (Invitrogen, United States).

### 2.8. Luciferase Reporter Assay

The DNA fragments containing rs7536272 A allele or G allele were amplified by using specific primers and then the products were cloned into pGL3 vector (Promega). Similarly, the cDNA of *SP1* was amplified and cloned into pCDNA3.1 (+) vector (Genomeditech) to construct *SP1* overexpression (*SP1* OE) plasmid. Next, all plasmids were confirmed by Sanger sequencing. A total of 2 × 10^5^ HEI‐OC1 cells were seeded onto 24‐well plates before transfection. The luciferase reporter plasmids were cotransfected with pRL‐TK vector (Promega) by using the Lipofectamine 2000 (Invitrogen, United States). To evaluate the effect of *SP1* on *PIK3R3* transcriptional activity, luciferase reporter plasmids were transfected with *SP1* OE or *SP1* negative control (*SP1*‐NC) plasmid. The Renilla‐Firefly luciferase activity was measured using the Dual‐Luciferase Reporter Assay (Promega).

### 2.9. Western Blotting

Proteins were extracted from the cells with the RIPA lysis buffer (Thermo Fisher, United States). Protein concentration was further measured using BCA assay kit (Beyotime Biotechnology, China). Proteins were separated using 10% SDS‐PAGE (Epizyme Biotech, China) and then transferred to PVDF membrane (Millipore, United States). After blocking with 5% skim milk in TBST, membranes were incubated with primary antibodies and then incubated with HRP‐conjugated secondary antibody (1:5000 ABclonal). Protein bands were visualized using the ECL chemiluminescence reagent (Millipore, United States). The immunoblot results were calculated using the ImageJ software (NIH) and then normalized to *GAPDH*.

Dilution of primary antibodies was as follows: *anti-PI3K* antibody (1:1000, Cell Signaling Technology), *anti-p-PI3K* antibody (1:1000, Cell Signaling Technology), *anti-AKT* antibody (1:1000, Cell Signaling Technology), *anti-p-AKT* antibody (1:2000, Cell Signaling Technology), *anti-LC3B* antibody (1:1000, Sigma‐Aldrich), *anti-SP1* antibody (1:1000, Abcam), *anti-caspase-3* antibody (1:2000, Abcam), *anti-Bax* antibody (1:1000, ABclonal), *anti-Bcl2* antibody (1:1000, ABclonal), *anti-PIK3R3* antibody (1:1000, Bioss), and *anti-GAPDH* antibody (1:1000, Abcam).

### 2.10. Cell Viability Assay

HEI‐OC1 cell viability was assessed using a Cell Counting Kit‐8 (CCK‐8) (Vazyme, Nanjing, China). HEI‐OC1 cells were transfected with si‐NC, si‐*SP1*, or si‐*SP1* plus *PIK3R3*‐OE plasmids. Cells were inoculated into 96‐well plates (5000 cells per well) and incubated overnight under 33°C in 10% CO_2._ Subsequently, 10 *μ*L of CCK‐8 reagent was added to each plate of the 0, 24, 48, and 72 h groups, followed by incubation for 2 h at 33°C. The absorbance at 450 nm was measured with a multimode microplate reader (Bio Tek Instruments, United States).

### 2.11. Statistical Analysis

IBM SPSS Statistics (Version 23.0; IBM, Chicago, Illinois, United States) was employed for statistical analysis. The data were summarized as mean ± standard deviation (SD) or standard error of the mean (SEM). Two‐tailed *χ* ^2^ goodness of fit test was performed to determine the Hardy–Weinberg equilibrium (HWE) in the controls. Two‐tailed *χ* ^2^ test was used to determine the frequency distribution of demographic characteristics and genotype of studied SNPs. Two‐tailed Student′s *t*‐test was used to determine the differences between groups. One‐way analysis of variance (ANOVA) along with Tukey′s multiple comparison test was used for multigroup comparisons. The association between the genotype and NIHL risk was determined via calculating adjusted odds ratio (OR) and 95% confidence interval (CI). A *p* value of less than 0.05 was statistically significant.

## 3. Results

### 3.1. Basic Features of Subjects

This case‐control study of 1355 subjects involved 688 diagnosed NIHL patients and 667 controls. The detailed descriptions of characteristics of subjects were presented in Table 1. Totally, 93.73% (1270) were males, and 6.27% (85) were females. The BHFTA of NIHL patients was significantly higher than that of the control group (*p* < 0.001). There were no statistical differences in the distribution of age, gender, noise exposure time, noise exposure intensity, smoking and drinking status, and CNE between the NIHL case and control subjects (*p* > 0.05).

**Table 1:** Demographic characteristics of NIHL patients and control subjects.

Variables	Cases (*n* = 688)		Controls (*n* = 667)		*p*
	*n*	%	*n*	%	
Age (years)					0.668^a^
Mean ± SD	39.81 ± 7.87		39.92 ± 7.96		0.799^b^
< 35	183	26.60	178	26.69	
35–45	343	49.85	319	47.83	
> 45	162	23.55	170	25.49	
Gender					0.680^a^
Male	643	93.46	627	94.00	
Female	45	6.54	40	6.00	
Work time with noise (years)					0.868^a^
Mean ± SD	18.06 ± 8.96		17.65 ± 8.80		0.389^b^
≤ 16	328	47.67	321	48.13	
> 16	360	52.33	346	51.87	
Smoking status					0.115^a^
No	424	61.63	383	57.42	
Yes	264	38.37	284	42.58	
Drinking status					0.908^a^
No	261	37.94	251	37.63	
Yes	427	62.06	416	62.37	
High‐frequency hearing threshold (dB)					< 0.001^a^
Mean ± SD	37.32 ± 12.38		15.80 ± 4.72		< 0.001^b^
≤ 26	74	10.76	667	100.0	
> 26	614	89.24	0	0.0	
Noise exposure level (L_Aeq, 8 h_, dB(A))					0.371^a^
Mean ± SD	87.82 ± 7.67		88.32 ± 7.23		0.224^b^
≤ 85	270	39.24	242	36.28	
86–91	117	17.01	108	16.19	
≥ 92	301	43.75	317	47.53	
CNE (dB(A)·year)					0.347^a^
Mean ± SD	99.69 ± 7.82		100.08 ± 7.30		0.357^b^
≤ 95	180	26.16	153	22.94	
96–101	188	27.33	184	27.59	
≥ 102	320	46.51	330	49.48	

^a^Two‐sided *χ* ^2^ test.

^b^Student′s *t*‐test.

### 3.2. SNP rs7536272 Located in PIK3R3 Was Significantly Associated With NIHL

The basic information of the eight SNPs located in the four genes was presented in Table 2. Some of the samples in this study failed to achieve successful genotype sequencing. As shown in Table S1, by further comparing the baseline indicators, including age, gender, noise exposure time, noise exposure intensity, and smoking and drinking status, as well as CNE between the successful classification group and the failed classification group, we found that there were no significant differences in all baseline indicators between the two groups (*p* > 0.05). The results suggest that the failure of genotype sequencing in the samples is due to random detection factors. The baseline of the two groups was balanced and comparable, and there was no selection bias, which did not affect the results of the genetic association analysis. The genotype distribution of all 8 SNPs included here displayed no deviation from HWE (*p* > 0.05). SNP rs7536272 in *PIK3R3* demonstrated significant differences in the frequencies of genotypes between the NIHL case and control subjects (*p* = 0.036). However, there were no significant differences in genotype frequencies between the NIHL group and the control group for the other seven SNPs (*p* > 0.05).

**Table 2:** General information of studied SNPs and Hardy–Weinberg test.

No.	Gene	dbSNP no.	Chromosome	Functional consequence	Allele A1/A2	MAF		*p* for HWE^b^	Case	Control	*p* ^c^
						Database^a^	Control		A1A1/A1A2/A2A2		
1	*AKT1*	rs2494732	14:104772855	Intron	C/T	0.266	0.274	0.655	317/270/59	330/255/45	0.305
2	*AKT1*	rs2494752	14:104797271	Promoter	C/T	0.295	0.274	0.827	302/274/49	332/247/48	0.243
3	*AKT1*	rs2498786	14:104796031	Promoter	C/G	0.207	0.194	0.515	386/217/33	405/201/21	0.159
4	*PIK3CA*	rs7651265	3:179175241	Intron	A/G	0.102	0.090	0.665	532/95/5	528/102/6	0.843
5	*PIK3R1*	rs706713	5:68226894	Exon	T/C	0.325	0.294	0.523	340/255/40	323/260/59	0.129
6	*PIK3R1*	rs1550805	5:68287979	Intron	C/T	0.101	0.091	0.426	534/97/4	532/103/7	0.618
7	*PIK3R1*	rs3730089	5:68292320	Exon	G/A	0.166	0.190	0.431	424/191/18	417/203/20	0.782
8	*PIK3R3*	rs7536272	1:46177421	Promoter	A/G	0.373	0.321	0.058	218/254/71	278/293/54	0.036

*Note:* A1 represents the major allele, and A2 represents the minor allele.

^a^Data from NCBI dbSNP.

^b^ *p* value of Hardy–Weinberg test.

^c^Two‐sided *χ* ^2^ test.

### 3.3. SNP rs7536272 G Allele Played an Underlying Risk Role in NIHL

As shown in Table 3, SNP rs7536272 GG genotype showed a significantly elevated impact on the risk of NIHL, compared with the AA genotype (*p* = 0.010, adjusted OR = 1.68, 95% CI, 1.13–2.50). Compared with the AA/AG combination genotypes, the subjects with the GG genotype seemed to be more sensitive to NIHL risk in recessive effect (*p* = 0.015, adjusted OR = 1.60, 95% CI, 1.10–2.32). In addition, the carriers of the G allele had a significantly higher risk of NIHL than those with the A allele (*p* = 0.024, adjusted OR = 1.22, 95% CI, 1.03–1.45).

**Table 3:** Genotype frequency distributions of SNP rs7536272 in NIHL cases and controls and the association with NIHL.

Genetic models	Genotypes/alleles	Cases (*n* = 688)		Controls (*n* = 667)		*p* ^a^	Adjusted OR (95% CI)^b^	*p* ^b^
rs7536272		*n* = 543	%	*n* = 625	%			
Codominant	AA	218	40.15	278	44.48	0.036	1.00 (ref)	
	AG	254	46.78	293	46.88		1.11 (0.87–1.42)	0.404
	GG	71	13.08	54	8.64		1.68 (1.13–2.50)	0.010
Dominant	AA	218	40.15	278	44.48	0.135	1.00 (ref)	
	AG + GG	325	59.85	347	55.52		1.20 (0.95–1.51)	0.128
Recessive	AA + AG	472	86.92	571	91.36	0.014	1.00 (ref)	
	GG	71	13.08	54	8.64		1.60 (1.10–2.32)	0.015
Alleles	A allele	690	63.54	849	67.92	0.026	1.00 (ref)	
	G allele	396	36.46	401	32.08		1.22 (1.03–1.45)	0.024

^a^Two‐sided *χ* ^2^ test.

^b^Adjusted for age, gender, smoking and drinking status in the logistic regression model.

We further performed stratified analysis to explore the interactions of rs7536272 with individual and/or environmental factors in recessive effect which were displayed in Table 4. Compared with the AA/AG combined genotypes, male subjects carrying the GG genotype had a 1.69‐fold higher risk of developing NIHL (*p* = 0.008, adjusted OR = 1.69, 95% CI, 1.15–2.49). Comparable elevated risks were also found among the subjects aged less than 35 years (*p* = 0.030, adjusted OR = 2.44, 95% CI, 1.09–5.44) and 35–45 years (*p* = 0.036, adjusted OR = 1.77, 95% CI, 1.04–3.01). Nonsmoking and nondrinking subjects carrying the rs7536272 GG genotype were more sensitive to NIHL. In addition, the interactions between gene‐environment factors revealed that the rs7536272 GG genotype significantly elevated the risk of NIHL in the groups of noise exposure level ≤ 85 dB(A) and CNE ≤ 95 dB(A)·year.

**Table 4:** Stratified analysis of the association between rs7536272 and NIHL in a recessive model.

Variables	AA/AG (case/control)		GG (case/control)		*p* ^a^	Adjusted OR (95% CI)^b^	*p* ^b^
	*n*	%	*n*	%			
Gender							
Male	437/535	40.05/49.04	69/50	6.32/4.58	0.007	1.69 (1.15–2.49)	0.008
Female	35/36	45.45/46.75	2/4	2.60/5.19	0.744	0.55 (0.09–3.30)	0.513
Age (years)							
< 35	121/156	39.54/50.98	19/10	6.21/3.27	0.025	2.44 (1.09–5.44)	0.030
35–45	234/278	40.63/48.26	38/26	6.60/4.51	0.039	1.77 (1.04–3.01)	0.036
> 45	117/137	40.91/47.90	14/18	4.90/6.29	0.805	0.89 (0.42–1.88)	0.762
Smoking status							
No	295/340	41.61/47.95	44/30	6.21/4.23	0.034	1.68 (1.03–2.74)	0.039
Yes	177/231	38.56/50.33	27/24	5.88/5.23	0.195	1.44 (0.80–2.59)	0.228
Drinking status							
No	178/218	40.36/49.43	28/17	6.35/3.85	0.028	1.93 (1.02–3.66)	0.044
Yes	294/353	40.44/48.56	43/37	5.91/5.09	0.160	1.40 (0.88–2.24)	0.155
Work time with noise (years)							
≤ 16	217/278	39.38/50.45	32/24	5.81/4.36	0.058	1.74 (0.99–3.05)	0.054
> 16	255/293	41.33/47.49	39/30	6.32/4.86	0.117	1.51 (0.91–2.51)	0.108
Expose level with noise (dB)							
≤ 85	178/203	41.11/46.88	33/19	7.62/4.39	0.023	2.05 (1.12–3.75)	0.020
86–91	84/91	41.79/45.27	14/12	6.97/5.97	0.578	1.57 (0.64–3.85)	0.321
≥ 92	210/277	39.33/51.87	24/23	4.49/4.31	0.295	1.38 (0.76–2.52)	0.295
CNE (dB(A)·year)							
≤ 95	105/128	39.18/47.76	24/11	8.96/4.10	0.009	2.81 (1.31–6.05)	0.008
96–101	151/157	44.15/45.91	19/15	5.56/4.39	0.448	1.34 (0.65–2.75)	0.431
≥ 102	216/286	38.71/51.25	28/28	5.02/5.02	0.318	1.35 (0.77–2.35)	0.291

^a^Two‐sided *χ* ^2^ test.

^b^Adjusted for age, sex, and smoking and drinking status in the logistic regression model.

### 3.4. SNP rs7536272 G Allele Significantly Increased the BHFTA Levels of the Subjects

Additionally, we analyzed the relationship between rs7536272 and BHFTA level and found that the subjects with a GG genotype were associated with higher sensitivity to noise in contrast to the AA and/or AG genotypes (Figure 1a). In addition, we observed that individuals carrying the rs7536272 AA/AG genotypes had lower BHFTA levels compared with those with the GG genotype (Figure 1b).

**Figure 1:**
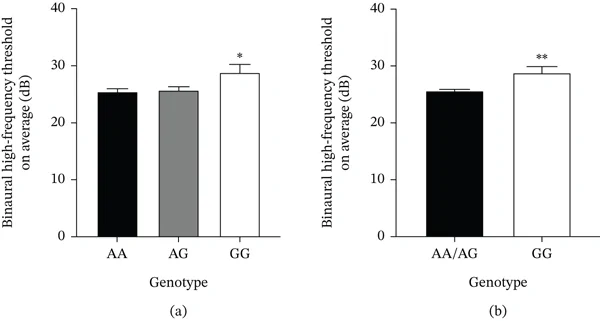
Comparison of binaural high‐frequency threshold on average (BHFTA) in carriers with different genotypes of rs7536272. (a) Comparison of BHFTA in subjects with AA, AG, and GG genotypes. (b) Comparison of BHFTA in subjects carrying AA/AG or GG genotypes. Data are presented as mean ± SEM.  ^∗^ *p* < 0.05, compared with the AA genotype.  ^∗∗^ *p* < 0.01, compared with the AA/AG genotypes.

### 3.5. SNP rs7536272 Regulated the PIK3R3 Expression in an Allele‐Specific Manner

To explore if rs7536272 regulates *PIK3R3* expression, we performed qRT‐PCR analysis to determine the mRNA expression of *PIK3R3* among the subjects carrying different genotypes. Firstly, we measured the expression of *PIK3R3* in 91 NIHL cases and 72 normal hearing controls. The genotype and allele frequency distributions of 91 NIHL cases and 72 controls concerning the SNP rs7536272 were presented in Table S2. The genotype and allele distribution of these subjects was consistent with that of the 688 cases and 667 normal controls. Next, we found that the expression of *PIK*3*R*3 was decreased in the NIHL cases compared with the control group (Figure 2a). Moreover, significant reductions in *PIK*3*R*3 expression appeared in the individuals with the GG genotype than the AA/AG combination genotypes carriers (Figure 2b).

**Figure 2:**
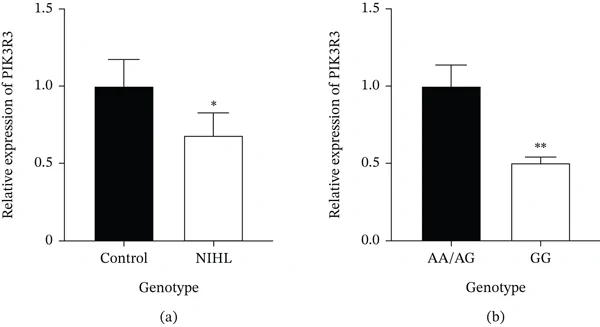
Analysis of *PIK3R3* expression in peripheral blood samples from NIHL and normal hearing subjects, as well as carriers of different genotypes. (a) Relative expression of *PIK3R3* in 91 NIHL cases and 72 controls was measured by qRT‐PCR. (b) *PIK3R3* expression was assessed by qRT‐PCR in subjects with AA/AG genotypes (*n* = 105) or GG genotype (*n* = 58). Data are presented as mean ± SEM.  ^∗^ *p* < 0.05,  ^∗∗^ *p* < 0.01, compared with the control group or the AA/AG genotypes.

### 3.6. SNP rs7536272 Allele‐Specifically Regulated the PIK3R3 Expression by Binding to SP1

Next, we would like to determine whether there were any potential transcription factors that interact with the genome sequence region including SNP rs7536272. We performed bioinformatics analysis and found that transcription factor *SP1* specifically binds to rs7536272‐containing DNA region (Figure 3a). Then, we evaluated whether *SP1* indeed regulates the expression of *PIK3R3* by binding to the rs7536272 A allele or G allele. By cotransfection of luciferase reporter plasmids containing rs7536272 A allele or G allele with *SP1* OE plasmid or control plasmid, we observed that the cells transfected with the A allele apparently increased the luciferase activities when compared with the G allele cotransfected with *SP*1 OE plasmid group (Figure 3b). There were significant differences between *SP*1 OE and control cells in rs7536272 A allele group, indicating that *SP*1 OE enhanced the luciferase activities of the cells. However, in cotransfected with *SP*1 control plasmid group, we found that there were no significant differences in the luciferase activity between both alleles of rs7536272. These findings state that *SP1* plays a crucial role in the regulation of rs7536272 on *PIK3R3* expression via binding to the specific allele of rs7536272.

**Figure 3:**
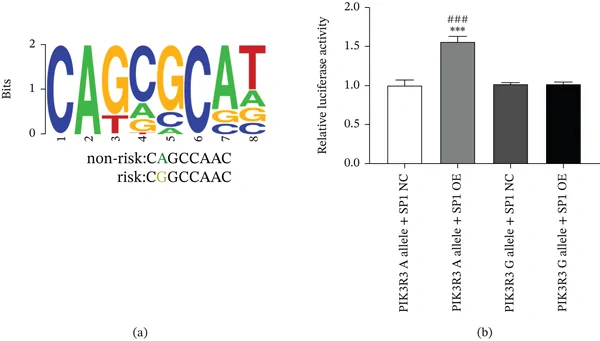
Rs7536272 regulated the binding of *SP*1 to the *PIK*3*R*3 promoter region. (a) Prediction of transcription factor *SP*1 interacts with rs7536272 by ChIPBase tool. (b) The effect of rs7536272 on *PIK*3*R*3 transcriptional activity was evaluated using luciferase reporter assay. *SP1* OE represents *SP*1 overexpression. Data are presented as mean ± SD from three independent experiments, each performed in triplicate (*n* = 3).  ^∗∗∗^ *p* < 0.001, compared with the *P* *I* *K*3*R*3 A allele + *S* *P*1 NC group. ^###^ *p* < 0.001, compared with the *P* *I* *K*3*R*3 G allele + *S* *P*1 OE group.

### 3.7. SP1 Suppresses Autophagy and Apoptosis in HEI‐OC1 Cells by Activating the PIK3R3‐Mediated PI3K/AKT Pathway

To address the biological effects of *SP1* on HEI‐OC1 cells, *SP1* was directed knocked down by means of three targeting siRNAs and transfected into the cells. The level of *SP1* protein was remarkably diminished when the cells incubated with the *si-SP1-1* and *si-SP1-3* (Figure 4a,b). When *SP1* was knocked down, the protein level of *PIK3R3* significantly decreased (Figure 4c). Given the central role of autophagy and apoptosis in determining cell fate [32], we next investigated the effect of *SP1* knockdown on these processes. *SP1* knockdown significantly inhibited the *PI3K/AKT* signaling pathway, indicated by the decreased phosphorylation levels of *PI3K* and *AKT* (Figure 4c,d). Concurrently, *SP1* deficiency promoted autophagy, as demonstrated by an elevated *LC3-II/LC3-I* ratio, and induced cell apoptosis, characterized by a decrease in the antiapoptotic protein *Bcl2* and an increase in the proapoptotic proteins *Bax* and *caspase-3* (Figure 4c,d).

**Figure 4:**
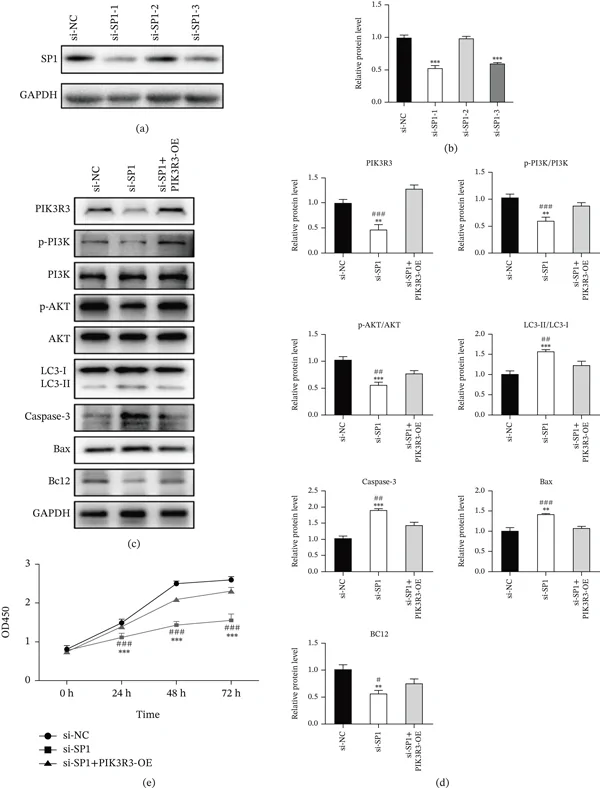
*SP*1 regulated autophagy, apoptosis, and cell viability in HEI‐OC1 cells through *PIK*3*R*3. (a) Western blotting was conducted to evaluate the efficiency of *SP*1 knockdown in HEI‐OC1 cells transfected with three specific siRNAs. (b) Quantitative analysis of the expression level of *SP*1 protein. (c) Western blotting was performed to detect the expression levels of autophagy‐ and apoptosis‐related proteins in HEI‐OC1 cells following *SP*1 knockdown and *PIK*3*R*3 overexpression. (d) Quantitative analysis of the expression levels of autophagy‐ and apoptosis‐related proteins. (e) Cell viability of HEI‐OC1 was measured by CCK‐8 assay at 0‐, 24‐, 48‐, and 72‐h posttransfection after *SP*1 knockdown and *PIK*3*R*3 overexpression. *PIK*3*R*3 OE represents *PIK*3*R*3 overexpression. Data are presented as mean ± SD from three independent experiments, each performed in triplicate (*n* = 3).  ^∗∗^ *p* < 0.01, and  ^∗∗∗^ *p* < 0.001, compared with the si‐NC group. ^#^ *p* < 0.05, ^##^ *p* < 0.01, ^###^ *p* < 0.001, compared with the si − *S* *P*1 + *P* *I* *K*3*R*3 − OE group.

To determine whether *PIK3R3* serves as the key functional downstream effector of *SP1*, we then performed a rescue experiment by overexpressing *PIK3R3* in *SP1*‐knockdown cells. Reintroducing *PIK3R3* into *SP1*‐knockdown cells could partially restore *PI3K/AKT* pathway activity and attenuate *SP1* knockdown‐induced enhancement of autophagy and apoptosis (Figure 4c,d). Furthermore, the CCK‐8 assay revealed that the knockdown of *SP1* led to a time‐dependent decrease in cell viability, which was significantly rescued by the overexpression of *PIK3R3* (Figure 4e). Collectively, these findings demonstrate that the transcription factor *SP1* functions to suppress autophagy and apoptosis in HEI‐OC1 cells, primarily by transcriptionally activating *PIK3R3* and thereby modulating the *PI3K/AKT* signaling axis.

## 4. Discussion

NIHL is a complex hearing disorder induced by the combined effect of environmental and genetic factors, exhibiting significant genetic hereditary susceptibility differences [10]. The occurrence of NIHL could affect the entire human lifecycle and alter the trajectory of hearing. For now, the identification of genetic polymorphism is a routine method for discovering high‐risk individuals developing NIHL. However, available susceptible polymorphisms by genetic analysis explain a small part of NIHL risk. Here, we conducted a case‐control study including 688 NIHL patients and 667 controls to explore more causal polymorphisms associated with NIHL.

The *PI3K/AKT* signaling pathway has been previously reported to play a crucial role in multiple biological function including gene expression, protein synthesis, cell survival, and apoptosis [12]. The abnormalities of *PI3K/AKT* signaling have previously been reported as an intense risk factor that can increase the susceptibility to NIHL ([14]). Recently, studies suggested that berberine as a quaternary ammonium hydroxide can weaken ROS production and apoptosis by activating *PI3K/AKT* signaling, thereby improving the survival of hair cells and spiral ganglion neurons and displaying otoprotective effect [33]. A mouse experiment revealed that calpain inhibitor MDL‐28170 reduced the functional defects and cochlear pathologies caused by noise by upregulating *PI3K/AKT* signaling [15]. In this study, by genetic analysis, we found that SNP rs7536272 located in *PIK3R3* was significantly associated with NIHL susceptibility. In detail, subjects with rs7536272 GG genotype were more sensitive to NIHL compared with the carriers with the AA/AG combined genotypes. Moreover, there were prominent synergistic effects between rs7536272 and several variables, showing that GG genotype carriers were more likely to develop NIHL than carriers of the AA/AG genotypes in the groups of males, aged less < 35 and 35–45 years old, nonsmokers and nondrinkers, noise exposure level ≤ 85 dB(A) and CNE ≤ 95 dB(A)·year. These findings were similar to previous research that SNPs influenced the personal health and led to the development and progression of diseases by interacting with environment factors [34]. Notably, the higher susceptibility of NIHL was observed in nonsmoking and nondrinking subjects carrying the GG genotype. Chronic smoking and alcohol exposure can preactivate endogenous antioxidant and DNA repair pathways (e.g., *Nrf2* signaling) and develop a sustained compensatory defense state [35]. Recent studies pointed out that the certain antioxidant genes were significantly upregulated in the smokers and drinkers, thereby preactivating the defense pathways to some extent [36–38]. Smoking and alcohol consumption can modify or even mask adverse phenotypes of specific risk genotypes by upregulating oxidative metabolism and stress repair pathways [39]. To sum up, we believe that the preadaptive mechanism triggered by lifestyle may counteract the genetic susceptibility and mask the adverse biological effects of the GG genotype. In contrast, individuals without smoking or alcohol exposure maintain a lower basal level of stress defense activation, which makes them more susceptible to the negative impact of genetic disadvantages. Nevertheless, this hypothesis remains speculative, and further experimental validation is required to clarify the regulatory mechanism.

In the past dozen years, even if many SNPs and genes associated with NIHL have been discovered, the applicability of these findings to clinical practice largely depends on their biological effects [40]. Previous reports illustrated that SNPs showed strong effects on gene function and disease phenotype by disturbing gene expression, mRNA stability, and/or protein conformation [30]. We first examined the expression of *PIK3R3* in blood samples using qRT‐PCR and observed that *PIK3R3* expression was significantly reduced in NIHL patients compared with normal controls. We then found that the subjects having the rs7536272 GG genotype showed lower *PIK3R3* levels than those carrying the AA/AG genotypes, accompanied by significantly higher levels of BHFTA. These findings emphasized that rs7536272 could allele‐specifically regulate *PIK3R3* expression and modify the risk of NIHL.

Emerging evidence has stated that SNP in the promoter region of gene has impact on the binding specificity of transcription factor by altering the DNA sequences of the transcription factor, thus influencing the regulation of gene expression [41]. To explore the potential effect of rs7536272 in *PIK3R3* regulatory region on NIHL, we predicted the transcription factors that may bind near rs7536272 locus and found that rs7536272 A > G generated a novel binding site for transcription factor *SP1*. *SP1* is one of members of the specificity protein‐like family and is the most active transcription activator [42, 43]. *SP1* was proven to be involved in many biological processes, particularly in cell growth, differentiation, apoptosis, and tumor initiation [43, 44]. Recently, studies confirmed that *SP1* bound to a variant (g.64943050 T > C) of *IGF1* and promoted granulosa cell proliferation [45]. SNP rs11573156 modified *PLA2G2A* expression by affecting the binding dynamics of *SP1* [46]. Similarly, we then carried out luciferase reporter assay in HEI‐OC1 cells to investigate the biological function of rs148582811, and discovered that the cells transfected with *SP1* OE plasmid significantly raised the luciferase activity compared with the negative control in cotransfected with rs7536272 A allele group. Further, we observed the luciferase activity was significantly increased in the cells when transfected with *SP1* OE plasmid with A allele, not the G allele. Our observations suggest that the A and G allele of rs7536272 has different regulatory functions. In particular, rs7536272 displays a regulatory effect on *PIK3R3* expression by influencing the binding efficiency of *SP1* to promoter region of *PIK3R3* in an allele‐specific manner.

Sufficient evidence has indicated that gene expression is a complicated process; in particular, transcription initiation displays a very significant role in regulating gene expression [47]. Notably, transcription factor actually is pretty important for transcription initiation in eukaryotes. *SP1* is a ubiquitous and widely expressed transcription factor in mammals and could bind to DNA and function as a transcription factor [48, 49]. By using luciferase reporter assay, we also found that *SP1* played a functional role in the regulation of rs7536272 on *PIK3R3* expression. After determining that *SP1* transactivates *PIK3R3* in an allele‐specific manner, we next investigated the functional consequences of this regulatory axis in HEI‐OC1 cells. Silencing the expression of *SP1* not only recapitulated the downregulation of *PIK3R3* observed with the risk G allele but also led to a significant suppression of the downstream *PI3K/AKT* signaling pathway, as indicated by reduced phosphorylation of *PI3K* and *AKT*. Furthermore, *SP1* deficiency promoted autophagy in cells, indicated by an elevated *LC3-II/LC3-I* ratio. Recent studies demonstrated that *SP1* is involved in the regulation of cell apoptosis and crucial to cellular functions [50], and we found that silencing *SP1* expression induced cell apoptosis, characterized by a significant decrease in *Bcl2* and concurrent increase in *Bax* and *caspase-3*. Importantly, restoration of *PIK3R3* expression in *SP1*‐silenced cells partially reversed these effects, including the reactivation of *PI3K/AKT* signaling and attenuation of autophagy and apoptosis. These findings support a model in which *PIK3R3* functions as a critical downstream effector of *SP1* in regulating cellular homeostasis. Therefore, our findings clearly reveal a pathway indicating that *SP1* inhibits the autophagy and apoptosis processes of HEI‐OC1 cells by maintaining the expression of *PIK3R3* and sustaining the activity of the *PI3K/AKT* pathway. Nevertheless, as loss‐of‐function experiments targeting *PIK3R3* were not performed in the present study, whether *PIK3R3* is strictly essential for mediating *SP1*‐dependent regulation of *PI3K/AKT* signaling, autophagy, and apoptosis remains to be fully elucidated. It is worth noting that, given the complexity of transcriptional regulatory networks and signaling crosstalk, it is possible that additional downstream effectors or parallel pathways may also contribute to the cellular effects driven by *SP1*. Therefore, although our findings support a hypothesis that *PIK3R3* plays a crucial role as a key downstream effector, further studies incorporating *PIK3R3* knockdown will be necessary to clarify its relative contribution and to define the mechanistic hierarchy within the *SP1*‐*PIK3R3*‐*PI3K/AKT* axis.

## 5. Conclusions

In summary, our present genetic association analysis reveals that SNP rs7536272 within *PIK3R3* was significantly correlated with NIHL; in particular, the G allele could elevate the risk of NIHL than the A allele. Moreover, rs7536272 modulates the expression of *PIK3R3* in an allele‐specific manner by binding to the *SP1* transcription factor. The *SP1*‐*PIK3R3* axis is implicated in the modulation of *PI3K/AKT* signaling and related cellular processes, including autophagy and apoptosis. These findings provide new insights into the genetic basis and potential molecular mechanisms underlying NIHL, whereas further studies are required to clarify the causal relationships and mechanistic hierarchy within this regulatory pathway.

## Funding

No funding was received for this manuscript.

## Ethics Statement

This study was performed in accordance with the Declaration of Helsinki, and it was approved by the Ethics Committee of Zhongda Hospital Affiliated to Southeast University (Approval No. 2020ZDSYLL150‐P01).

## Consent

Written informed consent was obtained from each individual who participated in this study.

## Conflicts of Interest

The authors declare no conflicts of interest.

## Supporting information


**Supporting Information** Additional supporting information can be found online in the Supporting Information section. Table S1: Demographic characteristics of successful classification samples and failed classification samples. Table S2: Genotype and allele frequency distributions of SNP rs7536272 in 91 NIHL cases and 72 controls.

## Data Availability

The data of this study are available from the corresponding authors upon reasonable request.
