# Supplementary material for: A Regulator SNP Affecting the Transcription Factor SP1 Binding Site in the PIK3R3 Gene Contributes to Noise‐Induced Hearing Loss Risk
Source: Hum Mutat. 2026 Oct 5;2026:7010704. doi: 10.1155/humu/7010704 (PMC13639393; doi:10.1155/humu/7010704)
Supplement: Supplementary file 1 [file HUMU-2026-7010704-s001.docx]

| **TABLE S1** Demographic characteristics of successful classification samples and failed classification samples | | | | | | | |
| --- | --- | --- | --- | --- | --- | --- | --- |
| Variables | Success samples (n = 1168) | |  | Failure samples (n = 187) | |  | *P* |
|  | n | % |  | n | % |  |  |
| Age (years) |  |  |  |  |  |  | 0.653^a^ |
| Mean ± SD | 39.87 ± 7.94 | |  | 39.86 ± 7.70 | |  | 0.988^b^ |
| < 35 | 306 | 26.20 |  | 55 | 29.41 |  |  |
| 35-45 | 574 | 49.14 |  | 88 | 47.06 |  |  |
| > 45 | 288 | 24.66 |  | 44 | 23.53 |  |  |
| Gender |  |  |  |  |  |  | 0.574^a^ |
| Male | 1093 | 93.58 |  | 177 | 94.65 |  |  |
| Female | 75 | 6.42 |  | 10 | 5.35 |  |  |
| Work time with noise (years) |  |  |  |  |  |  | 0.903^a^ |
| Mean ± SD | 17.90 ± 8.86 | |  | 17.58 ± 9.08 | |  | 0.644^b^ |
| ≤ 16 | 619 | 53.00 |  | 100 | 53.48 |  |  |
| > 16 | 549 | 47.00 |  | 87 | 46.52 |  |  |
| Smoking status |  |  |  |  |  |  | 0.920^a^ |
| No | 695 | 59.50 |  | 112 | 59.89 |  |  |
| Yes | 473 | 40.50 |  | 75 | 40.11 |  |  |
| Drinking status |  |  |  |  |  |  | 0.552^a^ |
| No | 723 | 61.90 |  | 120 | 64.17 |  |  |
| Yes | 445 | 38.10 |  | 67 | 35.83 |  |  |
| High frequency hearing threshold (dB) |  |  |  |  |  |  | < 0.001^a^ |
| Mean ± SD | 25.96 ± 14.39 | |  | 31.50 ± 12.76 | |  | < 0.001^b^ |
| ≤ 26 | 679 | 58.13 |  | 62 | 33.16 |  |  |
| > 26 | 489 | 41.87 |  | 125 | 66.84 |  |  |
| Noise exposure level (dB) |  |  |  |  |  |  | 0.910^a^ |
| Mean ± SD | 88.07 ± 7.34 | |  | 88.02 ± 8.23 | |  | 0.933^b^ |
| ≤ 85 | 444 | 38.01 |  | 68 | 36.36 |  |  |
| 86-91 | 193 | 16.52 |  | 32 | 17.11 |  |  |
| ≥ 92 | 531 | 45.46 |  | 87 | 46.52 |  |  |
| CNE (dB (A)·year) |  |  |  |  |  |  | 0.365^a^ |
| Mean ± SD | 99.90 ± 7.44 | |  | 99.77 ± 8.35 | |  | 0.840^b^ |
| ≤ 95 | 330 | 28.25 |  | 51 | 27.27 |  |  |
| 96-101 | 304 | 26.03 |  | 41 | 21.93 |  |  |
| ≥ 102 | 534 | 45.72 |  | 95 | 50.80 |  |  |
| ^a^Two-sided χ^2^ test. ^b^Students’ *t*-test. | | | | | | | |

| **TABLE S2** Genotype and allele frequency distributions of SNP rs7536272 in 91 NIHL cases and 72 controls | | | | | | | |
| --- | --- | --- | --- | --- | --- | --- | --- |
| Genetic models | Genotypes/Alleles | Cases (n = 91) | |  | Controls (n = 72) | | *P*^a^ |
| Codominant | AA | 34 | 37.36 |  | 36 | 50.00 | 0.037 |
|  | AG | 45 | 49.45 |  | 34 | 47.22 |  |
|  | GG | 12 | 13.19 |  | 2 | 2.78 |  |
| Dominant | AA | 34 | 37.36 |  | 36 | 50.00 | 0.106 |
|  | AG+GG | 57 | 62.64 |  | 36 | 50.00 |  |
| Recessive | AA+AG | 79 | 86.81 |  | 70 | 97.22 | 0.019 |
|  | GG | 12 | 13.19 |  | 2 | 2.78 |  |
| Alleles | A allele | 113 | 62.09 |  | 106 | 73.61 | 0.028 |
|  | G allele | 69 | 37.91 |  | 38 | 26.39 |  |
| ^a^Two-sided χ^2^ test. | | | | | | | |
